# Supplementary material for: Recent emergence of cephalosporin-resistant Salmonella Typhi in India due to the endemic clone acquiring IncFIB(K) plasmid encoding blaCTX-M-15 gene
Source: Microbiol Spectr. 2025 Apr 10;13(5):e00875-24. doi: 10.1128/spectrum.00875-24 (PMC12054180; doi:10.1128/spectrum.00875-24)
Supplement: Supplemental figures — Fig. S1 to S4. [file spectrum.00875-24-s0001.pdf]

Supplementary figures

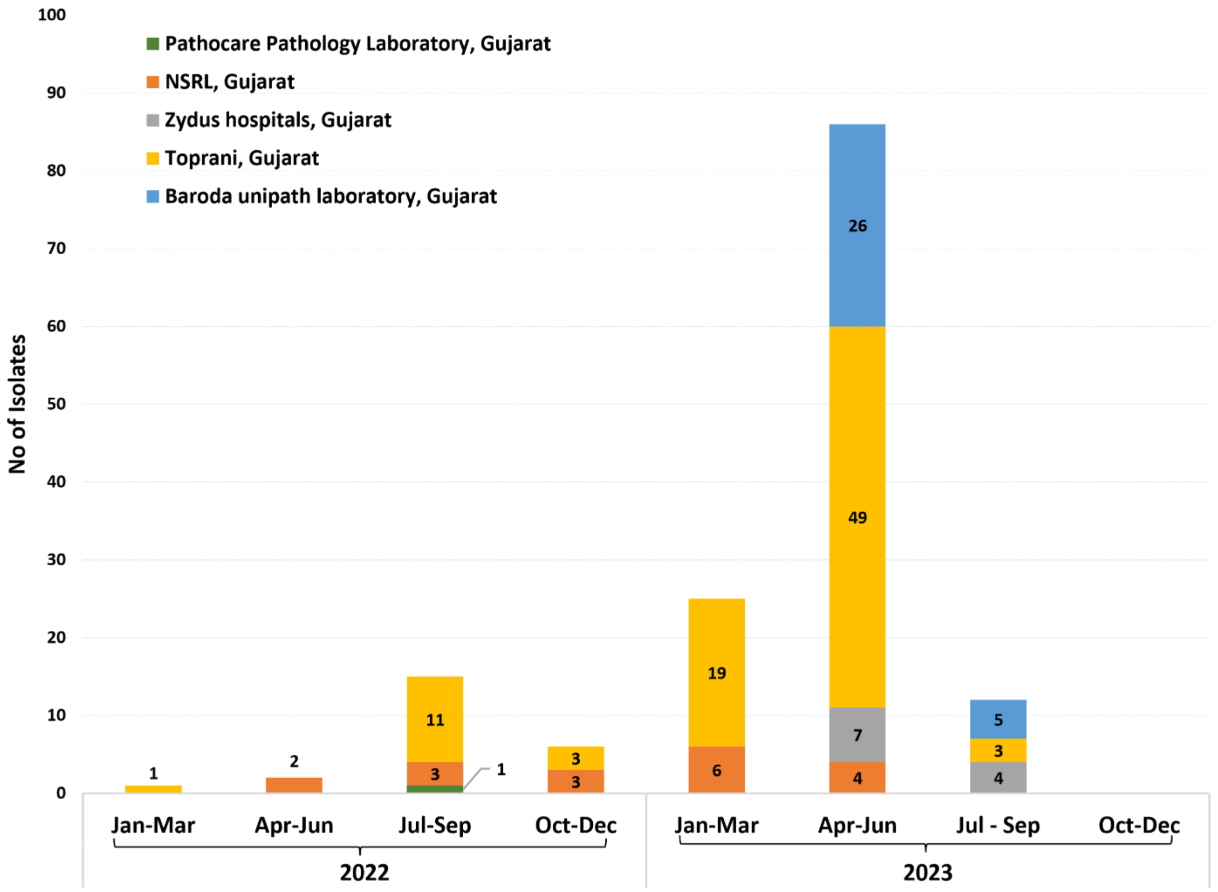

Suppl Fig: 1 Monthwise distribution of ceftriaxone resistant *S*. Typhi isolates received at the reference laboratory from April 2022 to March 2023

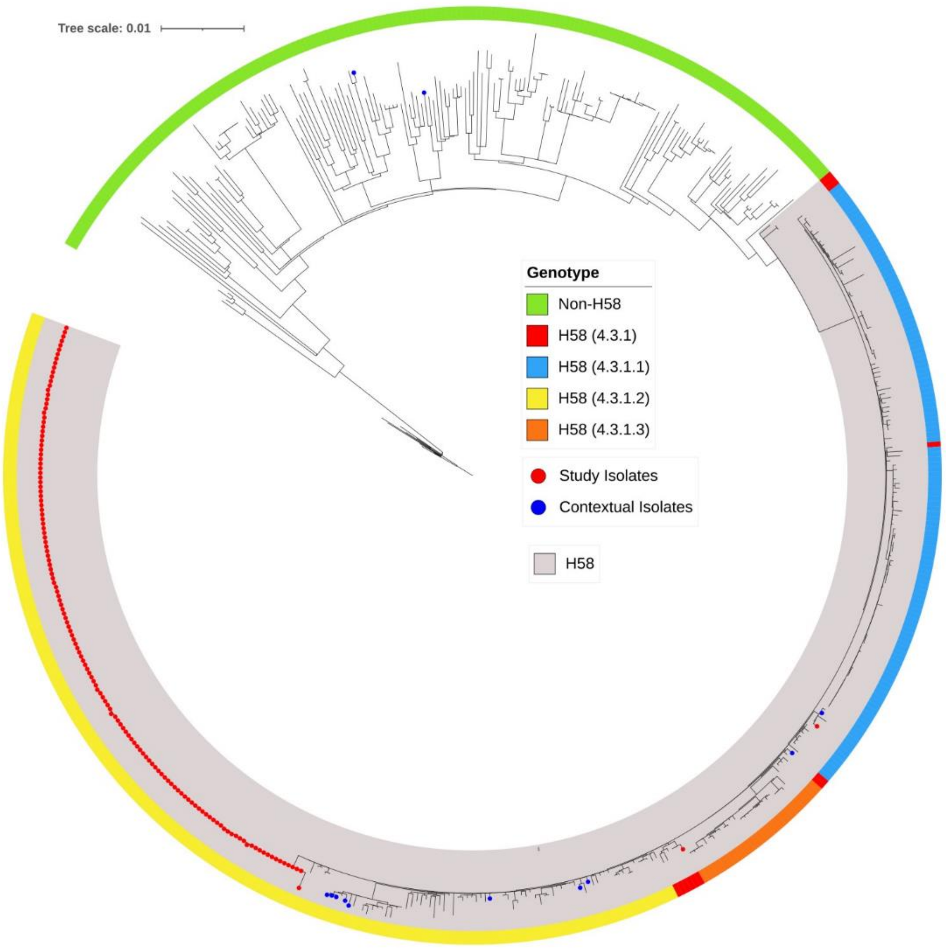

Suppl Fig 2: Phylogenetic structure of *S*. Typhi showing ceftriaxone resistant isolate sequences in a global context. Maximum likelihood core genome phylogenetic tree inferred for n=571 *S*. Typhi sequences (including n=142 from this study and n=11 contextual isolates). The tree was derived from 8980 core genome SNPs mapped against the reference genome of *S*. Typhi CT18 (Accession No: AL513382.1) using Snippy and rooted to the outgroup isolates (Genotype 0.1). Isolates belong to H58 lineage are shaded in Grey (#dcd4d4). Ring refer to major *S*. Typhi genotypes Non-H58, 4.3.1, 4.3.1.1, 4.3.1.2, 4.3.1.3 and respective colour codes for all the variables are given in the inset legend. Red-coloured branch symbols reflect the position of study isolates. Blue-coloured branch symbols reflect the position of contextual isolates The scale bar indicates substitutions per site. The tree was visualized and labeled using iTOL (https://itol.embl.de/).

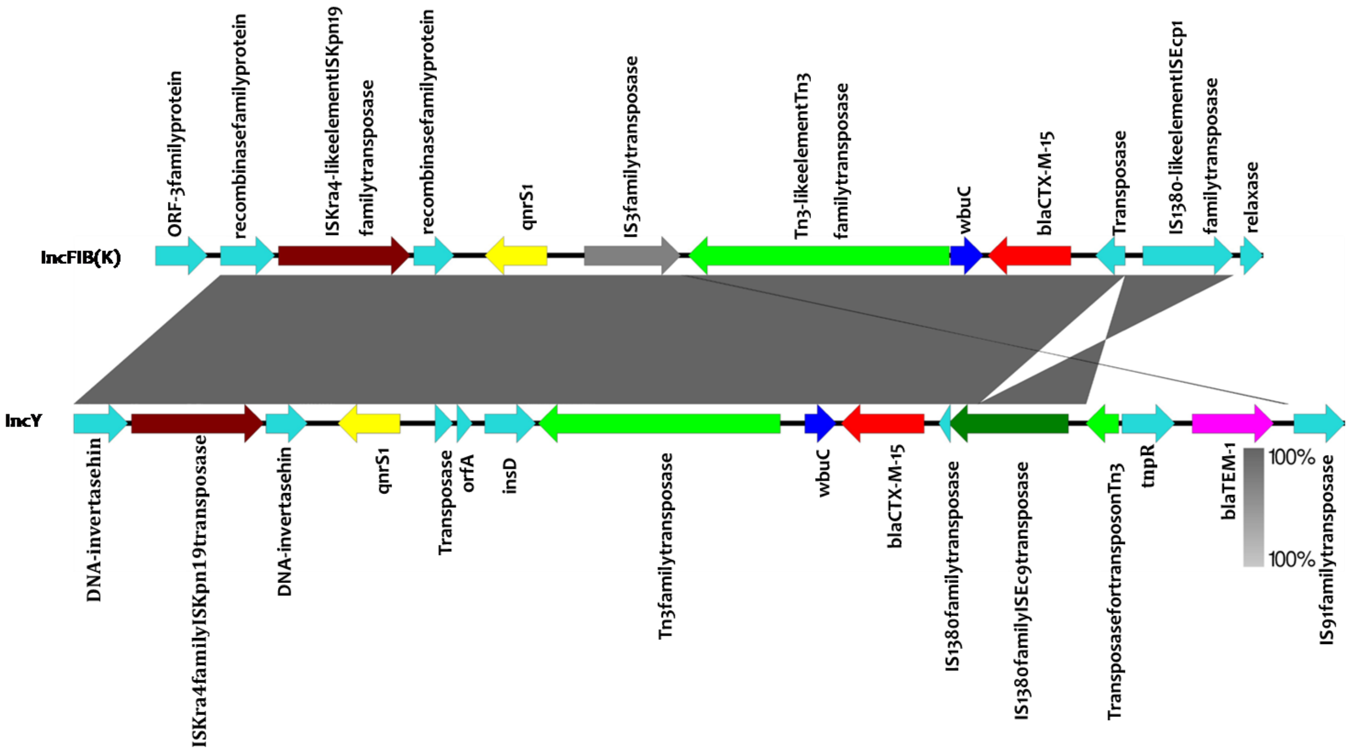

Suppl Fig 3: Genetic organization of IncFIB(K) plasmid (CP168964) and IncY plasmid isolated from *S*. Typhi was created using Easyfig BLASTn (https://mjsull.github.io/Easyfig/). The grey shading represents the sequence similarities and arrows represents the ORFs and their orientation.

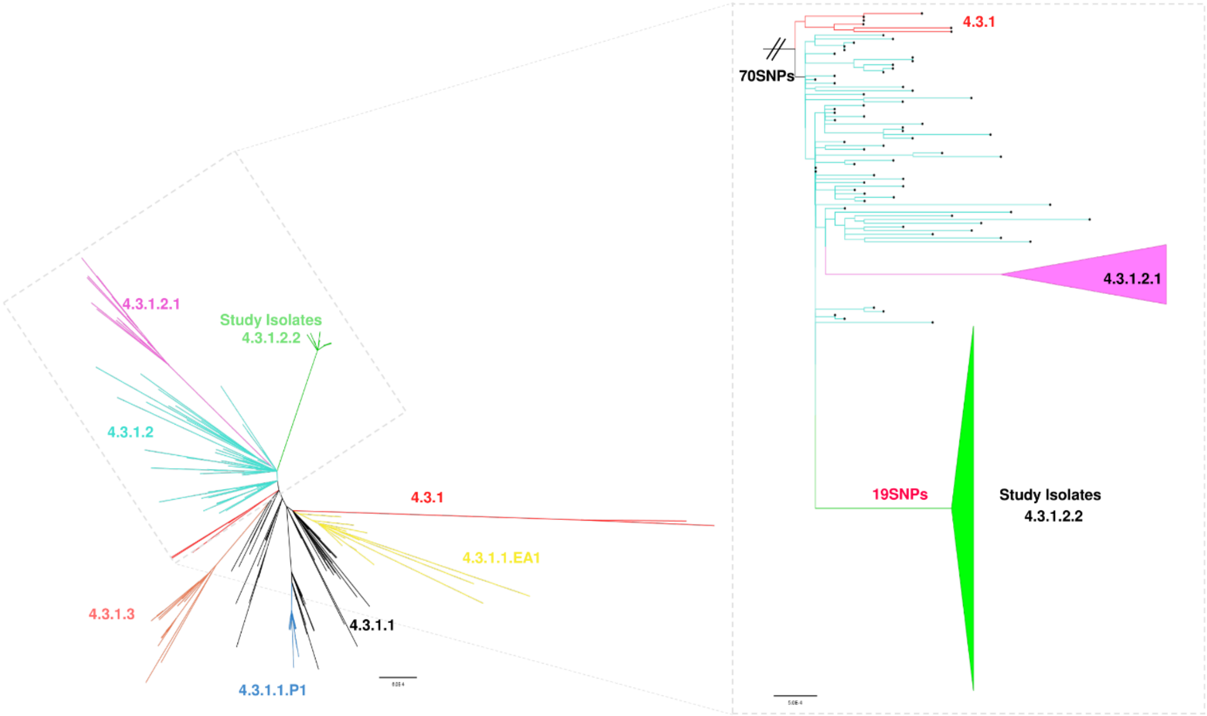

Suppl Fig 4: An unrooted maximum-likelihood phylogenetic tree of 247 global H58 Salmonella Typhi isolates, depicting the phylogenetic position of ceftriaxone-resistant isolates from Ahmedabad and Vadodara, India. Lineages are represented by distinct branch colors, with a higher-resolution diagram highlighting core SNP variations between branches. The ceftriaxone-resistant isolates (green branch) belong to genotype 4.3.1.2.2 and are separated by 19 SNPs from other 4.3.1.2 genotype.
